# Theranostic Options for Radioiodine-Refractory Differentiated Thyroid Carcinoma: Recent Advances, Challenges, and Road Ahead

**DOI:** 10.3389/fendo.2022.924841

**Published:** 2022-07-12

**Authors:** Swayamjeet Satapathy, Chandrasekhar Bal

**Affiliations:** Department of Nuclear Medicine, All India Institute of Medical Sciences, New Delhi, India

**Keywords:** radioiodine-refractory differentiated thyroid cancer, RAIR-DTC, theranostic, integrin binders, RGD, FAPI

## Abstract

Radioiodine-refractory differentiated thyroid cancer (RAIR-DTC), though uncommon, presents a considerable therapeutic challenge with poor long-term outcomes. Currently, tyrosine kinase inhibitors are the mainstay of treatment for advanced RAIR-DTC patients. However, these agents are associated with a multitude of adverse events with resultant deterioration in the quality-of-life of the patients. Targeted theranostic approaches with radiolabelled integrin binders and fibroblast activation protein- (FAP)-inhibitors seem to have a promising role in the management of such patients. This mini-review focuses on these novel theranostic strategies in RAIR-DTC, with emphasis on recent advances, existing challenges, and future directions.

## Introduction

Thyroid cancer, accounting for 586,000 cases worldwide, was ranked as the ninth most commonly incident cancer in the year 2020 (1). Differentiated thyroid cancers (DTCs), comprising ~90% of all thyroid cancers, usually have favourable long-term prognosis following surgery+/-radioiodine treatment, with mortality rates ranging from 0.3-0.5/100,000 population (1, 2). Nevertheless, the entity of radioiodine-refractory DTC (RAIR-DTC) presents a challenge to the treating physicians, with 10-year survival rate of ~10% from the time of detection of metastatic lesion(s) (3). The 2015 American Thyroid Association (ATA) guidelines classify RAIR-DTC into four categories: de-novo RAIR-DTC, i.e. the tumor never concentrated radioiodine; loss of ability to concentrate radioiodine; disease heterogeneity, i.e. some lesions concentrating radioiodine, while not others; and, lastly, progressive disease despite radioiodine concentration. Molecular imaging with 2-deoxy-2-[^18^F]fluoro-D-glucose-positron-emission-tomography/computed-tomography (2-[^18^F]FDG-PET/CT) is widely used to assess disease extent and progression in patients with RAIR-DTC. While asymptomatic indolent RAIR-DTC can be followed up with suppressive levothyroxine dose alone, symptomatic, progressive oligometastatic disease may benefit from directed therapies, namely, surgery, external radiotherapy, or thermal ablation. The major challenge lies with symptomatic, rapidly progressive, inoperable locally advanced/widely metastatic RAIR-DTC, which requires systemic therapies (4). Treatment options in this setting have been so far limited to only three tyrosine kinase inhibitors (TKIs) approved by the Food and Drugs Administration (FDA).

## Role of Tyrosine Kinase Inhibitors

The use of multikinase inhibitors has proven to be an effective strategy in the treatment of RAIR-DTC due to their action on the PI3K/Akt/mTOR- and MAPK-signaling pathways (5). However, given their toxicity profile, their use should be considered only for symptomatic, rapidly progressive, inoperable locally advanced/widely metastatic RAIR-DTC (4).

Sorafenib, an inhibitor of *VEGFR*, *PDGFR*, *cKIT*, and *RET*, was first approved for this indication based on the results of the DECISION trial (5). The trial randomized 417 patients to sorafenib or placebo, and reported a progression-free survival (PFS) benefit of 5.0 months. However, objective response rate (ORR) with sorafenib was modest at only 12.2% (6).

The results of the SELECT trial led to the approval of lenvatinib in RAIR-DTC. Lenvatinib inhibits *FGFR*, *VEGFR*, *PDGFR*, *cKIT*, and *RET*, and is administered orally at a dosage of 24 mg once daily in adults (5). The SELECT trial reported a significant PFS benefit of 14.7 months, as well as a remarkable ORR of 64.8% (7).

Cabozantinib is the latest drug that has been approved for RAIR-DTC patients, who have progressed on up to two prior *VEGFR* inhibitors. The COSMIC-311 trial reported a PFS benefit of 9.1 months with cabozantinib arm while the ORR was 18%. The recommended dosage is 60 mg orally once daily in adults (8).

Despite these encouraging results, the use of TKIs is associated with a wide spectrum of adverse events (AEs), such as hypertension, mucocutaneous toxicities, prolonged QTc interval, raised liver enzymes, proteinuria, haematological toxicities, and pancreatitis. Further, patients with prior cardiovascular events, poorly-controlled hypertension, prolonged QTc interval, or baseline deranged organ function are ineligible for treatment with these drugs (4). In this scenario, there is an unmet need for novel, effective and safe therapeutic strategies for patients with RAIR-DTC.

## Theranostics in RAIR-DTC

**Figure 1:**
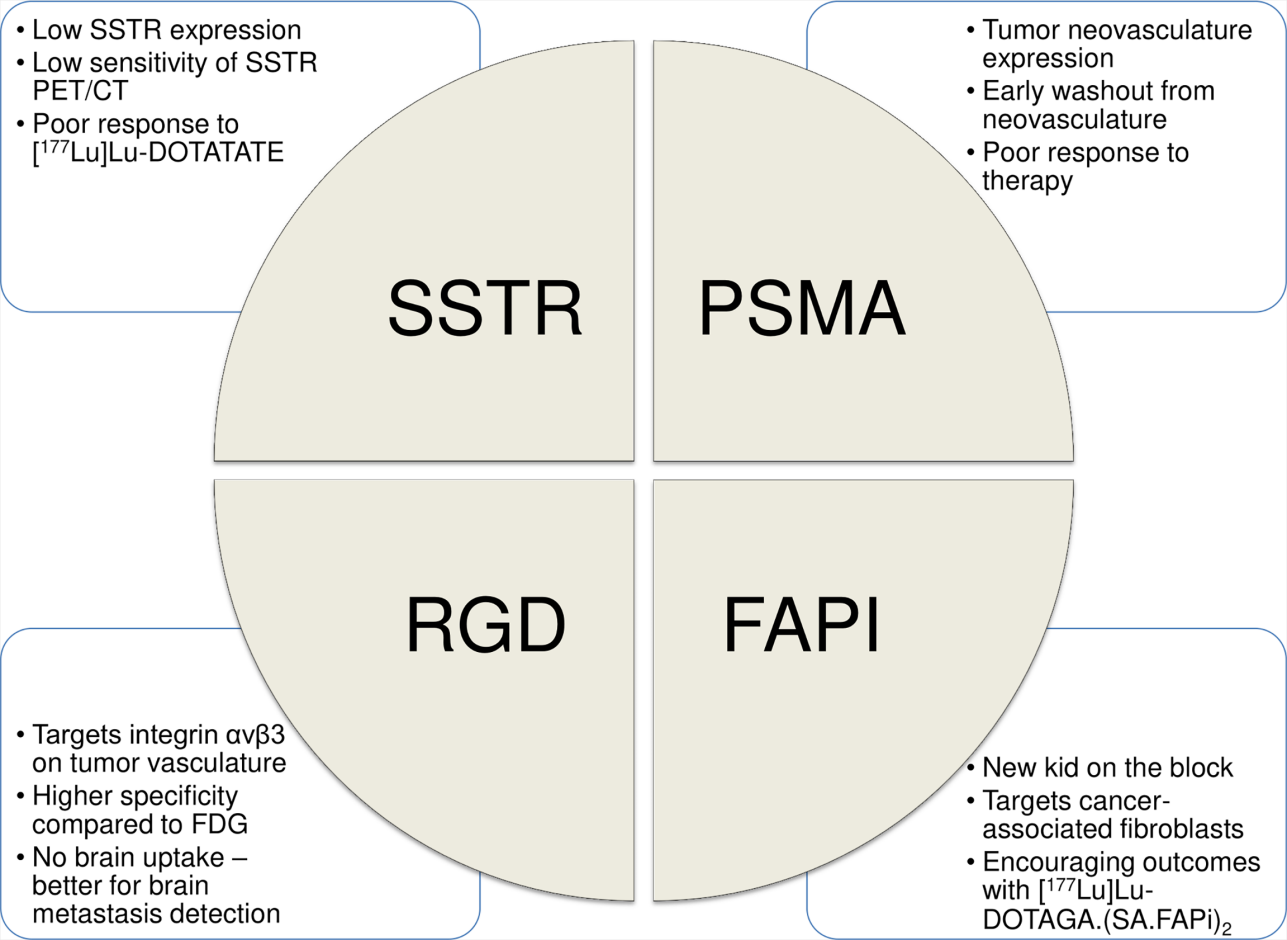
An overview of the currently available theranostic strategies for RAIR-DTC.

The field of “Theranostics” involves the combined approach of using same or similar radiopharmaceuticals for diagnostic and therapeutic purposes. In the past decade, use of theranostic pairs, targeted to somatostatin receptors (SSTRs) and prostate-specific membrane antigen (PSMA), has significantly altered the treatment paradigm in advanced neuroendocrine tumors (NETs) and prostate cancer (PCa), respectively. Herein, we explore the role of theranostics in RAIR-DTC, with emphasis on recent advances, existing challenges, and future directions. **Figure 1** enumerates the currently available theranostic strategies for RAIR-DTC.

### ^68^Ga/^177^Lu-Somatostatin Analogues

SSTRs are G-protein-coupled-receptors, overexpressed in well-differentiated NETs. Radiolabelled somatostatin analogues, namely, Gallium-68-(^68^Ga)-DOTANOC/DOTATOC/DOTATATE target these SSTRs, and have high sensitivity and specificity for the detection of well-differentiated NETs (9). The therapeutic counterpart, Lutetium-177-(^177^Lu)-DOTATATE was FDA-approved for advanced, progressive gastroenteropancreatic NETs (GEP-NETs) following the NETTER trial, which showed significant improvement in the PFS with [^177^Lu]Lu-DOTATATE (10). Subsequently, [^177^Lu]Lu-DOTATATE has also been tried successfully in the first-line setting in NETs (11, 12).

The expression of SSTRs in thyroid cells has been used to identify metastatic lesions and to select patients for peptide receptor radionuclide therapy (PRRT). One study enrolled 41 patients with progressive RAIR-DTC, and reported SSTR positivity in 24/41 patients (59%) compared to FDG positivity in 34/41 (83%) patients (13). In another study with 62 such patients, the authors reported SSTR-positive disease in 40/62 (65%) patients versus FDG-positive disease in 45/62 (72%) patients. Per-patient sensitivity and specificity of [^68^Ga]Ga-DOTANOC PET-CT was 78.4% and 100%, compared to 86.3% and 90.9%, respectively for 2-[^18^F]FDG-PET/CT (14). Overall, the sensitivity of SSTR-PET/CT was seen to be lower, and hence, unlikely to replace 2-[^18^F]FDG-PET/CT as the first-line imaging modality in RAIR-DTC.

Treatment-wise, PRRT with [^177^Lu]Lu-DOTATATE has been even less successful in RAIR-DTC. In a study comprising five RAIR-DTC patients with SSTR-positive disease on [^68^Ga]Ga-DOTATATE-PET/CT, the investigators administered 2–4 cycles of [^177^Lu]Lu-DOTATATE therapy (mean injected dose: 7.0± 0.7 gigabecquerels, GBq) at 12 weeks intervals. The results were poor with only one out of five patients having partial response, while three patients had disease progression (15). Similar results were reported in another study from India, where 6/8 (75%) patients with thyroglobulin-elevated negative iodine scintigraphy (TENIS) syndrome had radiological disease progression on [^177^Lu]Lu-DOTATATE. The reason for such modest responses could be the low degree of SSTR expression in RAIR-DTC, with corresponding high FDG avidity, which is suggestive of aggressive tumor behaviour (16). Notably, [^177^Lu]Lu-DOTATATE PRRT has also been tried with limited success in patients with advanced medullary thyroid cancer. Even in these cases, [^177^Lu]Lu-DOTATATE could mostly achieve stable disease, and not complete response or partial response (17, 18).

### ^68^Ga/^177^Lu-PSMA Ligands

PSMA is a type II transmembrane glycoprotein that is overexpressed in majority of the PCa cells, thereby making it an ideal target for diagnosis and therapy in PCa. PET/CT with the small molecule PSMA inhibitor, [^68^Ga]Ga-PSMA-11, has been shown to be useful in detecting involved sites in patients with metastatic PCa (19). In the landmark VISION trial, its therapeutic counterpart, [^177^Lu]Lu-PSMA-617, has been proven to improve overall survival, when added to standard-of-care, in patients with end-stage metastatic castration-resistant PCa (mCRPC) (20). Another phase-2 trial has also shown non-inferior outcomes with [^177^Lu]Lu-PSMA-617 compared to docetaxel in chemotherapy-naïve mCRPC (21).

Apart from the PCa cells, PSMA is also overexpressed in the endothelial cells of the neovasculature of various malignancies (19). This phenomenon has been sought to be explored in RAIR-DTC as well. In one of the earliest case series, comprising six patients with iodine-negative and FDG-positive metastasized DTC, the investigators reported PSMA-expressing disease in 5/6 (83%) patients. Of these, three patients had intense uptake on [^68^Ga]Ga-PSMA-11-PET/CT (22). However, in another series comprising nine patients with TENIS syndrome, the authors reported PSMA-positive disease in only 5/9 (56%) patients compared to FDG-positive disease in 8/9 (89%) patients. Further, out of a total of 14 lesions, [^68^Ga]Ga-PSMA-11-PET/CT detected less lesions compared to 2-[^18^F]FDG-PET/CT (9/14 versus 11/14, respectively) (23). Given the lower detection rates, the role of [^68^Ga]Ga-PSMA-11-PET/CT in RAIR-DTC lies primarily in patient selection for [^177^Lu]Lu-PSMA-617 therapy, and not for diagnostic purposes.

The first-in-human results of [^177^Lu]Lu-PSMA-617 in RAIR-DTC were reported in a series of five patients. Three patients were found to be eligible for [^177^Lu]Lu-PSMA-617, based on tracer uptake on [^68^Ga]Ga-PSMA-11-PET/CT, and of these, two patients underwent treatment. Two cycles of [^177^Lu]Lu-PSMA-617 (6.0 GBq per cycle) were administered to each patient at 6-11 weeks intervals. While one patient showed rapid disease progression one month later, the other patient showed partial response. Nevertheless, the response was not durable, with the patient eventually progressing seven months after treatment (24). The localization of PSMA expression in the neovasculature of RAIR-DTC instead of tumor cells as in PCa could potentially explain the relatively poor outcomes with [^177^Lu]Lu-PSMA-617 therapy in DTC compared to PCa.

### ^68^Ga/^177^Lu-RGD Analogues

Integrins are a group of heterodimer, transmembrane glycoprotein cell-adhesion molecules that are involved in cellular interactions and carcinogenesis. In particular, integrin αvβ3 is reported to be associated with tumor neoangiogenesis. It is also important in regulating the metastatic potential of tumor cells through its interactions with the extracellular matrix (25). Consequently, integrin αvβ3 is overexpressed in the tumor vasculature of various malignancies, and is an attractive target for diagnostic and therapeutic applications. More interestingly, in thyroid cancer, the αvβ3 integrin is reported to be overexpressed on both the tumor endothelial cells as well as the tumor cell surface (26, 27).

The Arg-Gly-Asp (RGD) tripeptide sequence has high affinity and specificity towards the integrin αvβ3 (28). Radiolabelled RGD analogues can, thus, be used for theranostic purposes in RAIR-DTC. This was demonstrated in a single-centre prospective study which enrolled 44 patients with RAIR-DTC for 2-[^18^F]FDG- and [^68^Ga]Ga‐DOTA‐RGD_2_-PET/CT studies. [^68^Ga]Ga‐DOTA‐RGD_2_-PET/CT detected 123 lesions, with an overall sensitivity, specificity and accuracy of 82.3%, 100%, and 86.4%, respectively. In contrast, 2-[^18^F]FDG-PET/CT detected 144 lesions, with overall sensitivity, specificity and accuracy of 82.3%, 50%, and 75%, respectively. Notably, [^68^Ga]Ga‐DOTA‐RGD_2_-PET/CT had similar sensitivity with much higher specificity compared to 2-[^18^F]FDG-PET/CT. This can be explained by the non-specific FDG accumulation in inflammatory/infective sites, in comparison to the more specific integrin localization in the tumor neovasculature (29). Similar results have also been reported with other RGD analogues, such as ^18^F-galacto-RGD, ^18^F-FPP-RGD2, and alfatide. Further, owing to the low background uptake in the normal brain tissue, radiolabelled RGD analogues have incremental role over 2-[^18^F]FDG-PET/CT in detecting brain metastases (30).

In the study by Parihar et al., the authors also reported that 82.1% patients positive on [^68^Ga]Ga‐DOTA‐RGD_2_-PET/CT had lesional radiotracer uptake higher than that of normal liver, and hence, suitable for potential treatment with [^177^Lu]Lu-RGD analogues (29). This is in stark contrast to the predominantly low-grade uptake with SSTR-analogues and PSMA-inhibitors. Further, [^177^Lu]Lu‐DOTA‐RGD_2_ is also reported to have favourable pharmacokinetics, as evident from biodistribution studies in mice with melanoma showing 80% clearance of radioactivity by 30 minutes after tracer administration, and 96% clearance by 72 hours. The rapid radiotracer clearance from the blood pool could potentially minimize the absorbed dose to the marrow. The tumor radioactivity retention was also good, with the highest tracer activity in the whole‐body at 72 hours being noted at the tumor site, i.e. 1.5% injected activity per gram of tissue (IA/g) (31). In a first-in-human study, Parihar et al. treated a 54-year-old female, having progressive TENIS syndrome with a single cycle of 5.5 GBq of [^177^Lu]Lu-DOTA‐RGD_2_. The patient had disseminated disease, and showed high tracer uptake on the baseline [^68^Ga]Ga‐DOTA‐RGD_2_-PET/CT. Four months after treatment, she experienced significant clinical as well as radiological response (32) (**Table 1**). Phase-1/2 trials with [^177^Lu]Lu-DOTA‐RGD_2_ are now warranted to determine the optimum radioactivity per cycle, while maximizing efficacy and minimizing toxicity.

**Table 1:** Salient features of [^177^Lu]Lu-DOTA-RGD_2_ and [^177^Lu]Lu-DOTAGA.(SA.FAPi)_2_ theranostic strategies.

Characteristic	[^177^Lu]Lu-DOTA-RGD_2_	[^177^Lu]Lu-DOTAGA.(SA.FAPi)_2_
Mechanism	Targets αvβ3 integrin in the tumor neovasculature	Targets fibroblast activation protein in the tumor stroma
Emission properties	Beta-emitter	Beta-emitter
Nature of molecule	Dimer	Dimer
Chelator	1,4,7,10-Tetraazacyclododecane-1,4,7,10-tetraacetic acid (DOTA)	1,4,7,10-tetraazacyclododececane,1-(glutaric acid)-4,7,10-triacetic acid (DOTAGA)
Lesion uptake for treatment eligibility	≥ liver (on ^68^Ga-DOTA-RGD_2_)	≥ pancreas (on ^68^Ga-DOTA.SA.FAPi)
Excretion	Renal	Hepatobiliary >> Renal
Target organ	Bladder wall	Colon
Whole-body effective half-life	~87% clearance by 24 hours, and ~96% by 72 hours (31)	~46 hours (~20% clearance by 24 hours, and ~30% by 72 hours) (33)
Tumor retention	~1.5% at 72 hours (31)	~1 – 4% at 72 hours (33)
Efficacy data	Single case report showing partial response (32)	Pilot study of 15 patients, partial response in 50% (34)

### ^68^Ga/^177^Lu-FAP Inhibitors

Fibroblast activation protein (FAP) is a type II transmembrane serine protease that is overexpressed in cancer-associated fibroblasts (CAFs), and plays a key role in modulating the tumor microenvironment, angiogenesis, and chemotherapy resistance. FAP is overexpressed in a variety of tumors, such as colon, pancreatic, ovarian, hepatocellular carcinoma, etc (35). Several radiolabelled small molecule FAP inhibitors (FAPIs), namely, [^68^Ga]Ga-FAPI-02, -04, -46, and -DOTA.SA.FAPi have been tried for imaging purposes in various malignancies (35–37). The lack of FAP expression in normal tissues results in a low background uptake with high image contrast. This has consequently led to [^68^Ga]Ga-FAPI-PET/CT outperforming 2-[^18^F]FDG-PET/CT in sensitivity and specificity for primary, nodal, and metastatic lesion evaluation across tumor types (38). Radiolabelled FAP inhibitors, therefore, present an interesting avenue for diagnostic and therapeutic applications in RAIR-DTC.

Fu et al. demonstrated intense FAPI uptake in local recurrent and distant metastatic sites in a patient with TENIS syndrome. In a subsequent case of RAIR-DTC, the same group detected additional metastatic lesions on [^68^Ga]Ga-FAPI-PET/CT, which were not positive on 2-[^18^F]FDG-PET/CT. This was attributed to the much better tumor-to-background ratio in [^68^Ga]Ga-FAPI-PET/CT compared to 2-[^18^F]FDG-PET/CT (39, 40). While adequately powered studies comparing the two modalities in RAIR-DTC are currently lacking, the results from these initial experiences as well as those extrapolated from other tumor types clearly favour an incremental role of [^68^Ga]Ga-FAPI-PET/CT in this setting.

In the field of therapeutics, ^177^Lu-labelled FAPI has witnessed considerable initial success. A major challenge with the initial small molecule FAPIs was their relatively lower tumor residence time owing to faster clearance. This was overcome by Moon et al. by developing a homodimeric system having two squaramide-conjugated FAP inhibitors connected by a central, bifunctional chelator (DOTAGA) to increase the tumor residence time, i.e. DOTAGA.(SA.FAPi)_2_. PET studies in six patients revealed significantly higher tumor uptake and longer tumor retention for [^68^Ga]Ga-DOTAGA.(SA.FAPi)_2_ compared to [^68^Ga]Ga-DOTA.SA.FAPi (41). Subsequent dosimetry studies also reported substantially higher median whole-body effective half-life (T_e_) (46.2 hours versus 23.1 hours, respectively), tumor T_e_ (86.6 hours versus 14 hours, respectively), and tumor absorbed dose (6.7 Gy/GBq versus 0.6 Gy/GBq, respectively) with [^177^Lu]Lu-DOTAGA.(SA.FAPi)_2_ in comparison to [^177^Lu]Lu-DOTA.SA.FAPi (33). Building on these initial encouraging results, the authors prospectively enrolled 19 RAIR-DTC patients, who had prior progression on sorafenib/lenvatinib. Fifteen/19 (79%) patients had intense tracer uptake (≥pancreatic uptake) on the baseline [^68^Ga]Ga-DOTAGA.SA.FAPi-PET/CT, and underwent treatment with 2-3 cycles of [^177^Lu]Lu- DOTAGA.(SA.FAPi)_2_ at 8 weeks intervals (cumulative activity, 8.2 ± 2.7 GBq). On follow-up, the patients experienced significant improvement in the performance scores and serum thyroglobulin levels, with no grade 3/4 AEs. Most notably, of the eight patients evaluated for morphological response, four (50%) achieved partial response and three (38%) had stable disease (disease control rate, 88%) (34) (**Table 1**). The results of this pilot study suggest [^177^Lu]Lu- DOTAGA.(SA.FAPi)_2_ to be a safe and effective strategy for the management of RAIR-DTC patients, particularly for those who have exhausted or are intolerable to the existing treatment options.

## Discussion

Diagnostic and treatment options in RAIR-DTC have come a long way over the last decade. While 2-[^18^F]FDG-PET/CT remains the modality of choice in the initial evaluation of RAIR-DTC patients, newer targeted approaches, such as [^68^Ga]Ga‐DOTA‐RGD_2_ and [^68^Ga]Ga-FAPI-PET/CT seem to have an incremental role. The ability to use these molecules for therapeutic purposes further adds to the armamentarium of treatment options in this setting. With encouraging results from pilot studies, these molecules hold considerable promise for patients with advanced RAIR-DTC.

Theranostic approaches have certain distinctive advantages over the current standard-of-care in advanced RAIR-DTC, i.e. TKIs. Given their non-specific nature of action, TKIs are associated with various AEs, affecting multiple systems (4, 5). In contrast, a targeted approach with radiotheranostics has limited off-site action, and hence, a high degree of safety margin, as evident from the preliminary studies (33, 34). Further, with the exception of lenvatinib, the existing TKIs mostly cause stable disease on follow-up (6–8). However, extrapolation of data from SSTR and PSMA theranostics in advanced NETs and mCRPC respectively, as well as results from the pilot studies with [^177^Lu]Lu-DOTA‐RGD_2_ and [^177^Lu]Lu- DOTAGA.(SA.FAPi)_2_ in RAIR-DTC, suggest higher partial response rates with the theranostic approaches (10, 20, 32, 34). This, in turn, can potentially result in increased PFS and overall survival for these patients, and the same remains to be validated in future studies.

Another major limitation with the currently approved TKIs is their high costs, especially in low-to-medium income countries. A prolonged duration of treatment with these agents causes significant financial burden on the patients, with many discontinuing treatment. Radionuclide therapy can, thus, prove to be an effective alternative for such patients. Since these therapies are administered at 2-3 monthly intervals, and up to a limited number of cycles, the financial burden on the patients could be relatively lower. This, coupled with their efficacy and safety, can potentially lead to improved quality-of-life for such patients. Interestingly, in a cost-consequence analysis for patients with pancreatic NETs, [^177^Lu]Lu-DOTATATE was associated with lower costs per progression-free month compared to the TKI, sunitinb (€2989 versus €5337, respectively) (42).

Despite the initial successes with theranostics in RAIR-DTC, few challenges remain. At the outset, SSTR and PSMA theranostics seem to have limited role here and only useful in selected individuals. In contrast, high-grade lesional uptake is noted in ~80% of patients on [^68^Ga]Ga‐DOTA‐RGD_2_ and [^68^Ga]Ga-FAPI-PET/CT, making these suitable for routine applications (29, 34). Nevertheless, human dosimetry data for [^177^Lu]Lu‐DOTA‐RGD_2_ is currently lacking, and its efficacy and safety remain to be validated in a patient cohort. Though [^177^Lu]Lu- DOTAGA.(SA.FAPi)_2_ has crossed these initial hurdles, few key problems persist. While dimerization of the FAPI molecule increased its intratumoral accumulation, it also prolonged its clearance from the blood pool, thereby potentially causing increased marrow toxicity. Although the initial results do not suggest major grade 3/4 AEs, the sample size is small and further studies are warranted to clearly demonstrate its safety. Further, the dimer FAPI molecule is predominantly excreted by the hepatobiliary route, thereby resulting in the colon being the target organ and receiving the highest absorbed dose (33, 34). Since significant radiotracer concentration is noted in the colon for the initial 48 hours, laxatives could be administered to accelerate its clearance and reduce toxicity. Another potential problem with FAPI theranostics could be that the targeting of the CAFs by the radiolabelled molecule may result in destruction of the tumor stroma only, and not tumor cells per se, owing to the low tissue path length of ^177^Lu beta particles.

In conclusion, novel theranostic strategies with RGD analogues and FAPI hold immense promise to alter the treatment paradigm in advanced RAIR-DTC. Adequately powered phase-2/3 trials are now required to generate further data on their efficacy and safety, including long-term outcomes. Targeted alpha therapy using these molecules could also be considered for refractory patients, and is an interesting topic for future research.

## Author Contributions

SS: Literature search, article selection, data extraction, manuscript writing. CB: Conception, literature search, article selection, data extraction, interpretation, manuscript refinement, and final approval. All authors contributed to the article and approved the submitted version.

## Conflict of Interest

The authors declare that the research was conducted in the absence of any commercial or financial relationships that could be construed as a potential conflict of interest.

## Publisher’s Note

All claims expressed in this article are solely those of the authors and do not necessarily represent those of their affiliated organizations, or those of the publisher, the editors and the reviewers. Any product that may be evaluated in this article, or claim that may be made by its manufacturer, is not guaranteed or endorsed by the publisher.
